# Novel Therapeutic Approaches to Psoriasis and Risk of Infectious Disease

**DOI:** 10.3390/biomedicines10020228

**Published:** 2022-01-21

**Authors:** Alfonso Motolese, Manuela Ceccarelli, Laura Macca, Federica Li Pomi, Ylenia Ingrasciotta, Giuseppe Nunnari, Claudio Guarneri

**Affiliations:** 1 Department of Clinical and Experimental Medicine, Section of Dermatology, University of Messina, Messina, Italy C/O A.O.U.P. “Gaetano Martino”, via Consolare Valeria, 1, 98125 Messina, Italy; alfonsomotolese93@gmail.com (A.M.); lauramacca7@gmail.com (L.M.); federicalipomi@hotmail.it (F.L.P.); 2 Department of Clinical and Experimental Medicine, Unit of Infectious Diseases, University of Catania, Catania, Italy C/O ARNAS “Garibaldi”, “Nesima” Hospital, via Palermo 636, 95122 Catania, Italy; manuela.ceccarelli@unict.it; 3 Department of Biomedical and Dental Sciences and Morphofunctional Imaging, Unit of Infectious Diseases, University of Messina, Messina, Italy C/O A.O.U.P. “Gaetano Martino”, via Consolare Valeria, 1, 98125 Messina, Italy; 4 Department of Biomedical and Dental Sciences and Morphofunctional Imaging, Section of Pharmacology, University of Messina, Messina, Italy C/O A.O.U.P. “Gaetano Martino”, via Consolare Valeria, 1, 98125 Messina, Italy; yingrasciotta@unime.it; 5 Department of Clinical and Experimental Medicine, Unit of Infectious Diseases, University of Messina, Messina, Italy C/O A.O.U.P. “Gaetano Martino”, via Consolare Valeria, 1, 98124 Messina, Italy; giuseppe.nunnari@unime.it; 6 Department of Biomedical and Dental Sciences and Morphofunctional Imaging, Section of Dermatology, University of Messina, Messina, Italy C/O A.O.U.P. “Gaetano Martino”, via Consolare Valeria, 1, 98125 Messina, Italy

**Keywords:** psoriasis, infections, HIV, HBV, HCV, tuberculosis, COVID-19, anti-TNF-*α*, antiIL-23, anti-IL-17, anti-IL12/23, anti-PDE4, anti-JAK

## Abstract

Psoriasis is a chronic immune-mediated skin and joint disease, with a plethora of comorbidities, characterized by a certain genetic predisposition, and a complex pathogenesis based on the IL-23/IL-17 pathway. There is no doubt that the patients affected by psoriasis are more susceptible to infections as well as that the risk of infection is higher in psoriatic subjects than in the general population. The advent of biotechnological agents on the therapeutic arsenal actually available for the treatment of moderate-to-severe patients, given the fact that the severity of the disease is a predictor of the level of infectious risk, has raised the question of whether these ‘new’ drugs could be considered a safer option and how they can be used in selected cases. Old and newer strategies in cases of chronic infectious conditions are reviewed under the light of clinical trials and other studies present in literature.

## 1. Introduction

Psoriasis is a chronic and recurrent inflammatory disease predominantly involving the skin [1]. It is characterized by well-defined, scaly, erythematous plaques, occasionally covered with silvery scales [1]. Lesions appear especially over the extensor surfaces, scalp, and lumbosacral region, but any skin surface can be involved [2]. Psoriasis is considered a common disease with an incidence of about 1–3% in the world population, and it affects men and women equally [3,4]. The pathogenesis of psoriasis involves a feed-forward mechanism of inflammation, including primarily the T-helper cell type 17 (TH17) and IL-23 pathway [1]. The dendritic cells, activated by numerous cytokines produced by keratinocytes, natural killer T-cells, and macrophages, secrete IL-12 and IL-23 inducing the differentiation of native T-cells to TH1 cells and the survival and proliferation of TH17 and TH22 cells [1,5,6]. This results in the production of several pro-inflammatory cytokines, such as IFNγ, TNFα and IL-22 [1], which are conducive to the proliferation of downstream keratinocyte, to the increased expression of angiogenic mediators and endothelial adhesion molecules, and to the infiltration of immune cells into lesional skin [7]. It is important to note that, as in multifactorial diseases, genetic factors have a critical role in the pathogenesis of psoriasis [5], and environmental factors can exacerbate the manifestations [5]. Psoriasis is associated with several comorbidities, including psoriatic arthritis cardiometabolic diseases, depression [5,8,9,10]; it is also associated with infectious diseases such as HIV, of which it is a revealing sign [11]. In fact, despite the availability of numerous systemic agents, choosing the right therapy might be challenging in certain categories of patients [6]. Psoriasis Area Severity Index (PASI) score is the most used tool to evaluate the severity of psoriasis and the outcome of the treatments [6]. During the last decades, the incidence of psoriasis increased and the deeper understanding of the pathogenesis led to a development of novel therapeutic approaches [1]. Tumor necrosis factor (TNF)-α antagonists totally changed the clinical history of the management of psoriasis and research interest is high in the newest biological drugs approved, especially drugs blocking IL-23/IL-17 [12]. While these therapies appeared to be effective in clearing cutaneous manifestations, their adverse event profiles frequently include infections [13]. The aim of our review is to analyze the new therapeutic approaches for the treatments of psoriasis, the potential risk of infections caused by these agents and the treatment strategies for patients with concomitants chronic infections.

## 2. Materials and Methods

Articles derived from the database Pubmed (https://ncbi.nlm.nih.gov/PubMed, accessed on 1 April 2021) and Embase (https://embase.com, accessed on 1 April 2021), published between 2018 and 2021. The keywords used were (“psoriasis”) AND (“infection”, “biologic”, “TNF-α inhibitors”, “IL-17 inhibitors”, “IL-12/23 inhibitors”, “IL-23 inhibitors”, “anti-PDE4”, “anti-JAK”). Only papers written in English were considered. Data were extracted and selected by two experts in the field of dermatology.

## 3. Discussion

### 3.1. Risk of Infection in Psoriasis

Infectious diseases are the second leading cause of death among psoriasis patients receiving therapies for moderate-to-severe disease [14]; however, the mechanism by which psoriasis predisposes to infection is still unclear [13]. According to some, patients with psoriasis have multiple risk factors for serious infections, such as immune dysregulation characterized by an increase of TNF and IL-17, and the use of systemic immunosuppressants [15,16]. The increased risk of infections caused by psoriasis is extensively documented in the literature [15,17,18,19]. As shown by Yiu et al. [18], there is a concrete risk of serious infection in psoriatic patients (20.5% per 1000 person-years) compared with those without psoriasis (16.5 per 1000 person-years), especially across the respiratory system and the soft-tissue/skin, which are the most common infection sites [18]. Hsu et al. [19] studied the association between psoriasis and the risk of infections in patients in the United States, showing that psoriatic patients are more prone to the risk of multiple infectious diseases, including viral infections, fungal infections, cellulitis, and infectious arthritis. The study also underlined that infections including pneumonia, septicemia, enterocolitis, meningitis, encephalitis, and peritonitis lead to the highest mortality rates [19]. In addition, it is important to also consider the significant impact of these serious infections on costs of care for patients: the study shows that the mean cost of care for patients with psoriasis and any serious infection is considerably higher (USD 13,291 6 USD 166) than for psoriatic patients that do not present such serious infections (USD 11,003 6 USD 96) [19]. To evaluate the risk of infection, it is important to consider the gravity of psoriasis. In fact, Takeshita et al. [15] found that the risk of having serious infection was higher for patients with moderate-severe psoriasis (49.5 per 10,000 person-years) compared with mild psoriasis (14.4 per 10,000 person-years). According to this study, psoriasis severity is a predictor of serious infection risk beyond traditional risk factors for infection [15]. In contrast, higher risks of opportunistic infections and herpes zoster were essentially limited to those patients receiving therapies for moderate-to-severe psoriasis and were associated with immunosuppressive therapies [15,20]. Moreover, the majority of the new systemic agents for psoriasis are immunosuppressive, which determine a singular treatment challenge in patients with psoriasis with chronic infections, such as tuberculosis, HIV, and hepatitis, because of their immunosuppressed profile [21]. Factors to consider when selecting systemic treatment during infections are summarized in Table 1.

### 3.2. Tubercolosis

Tuberculosis (TB) is the world’s most deadly infectious disease with 1.5 million deaths per year [22]. Once infected, the individual is at the highest risk of developing TB disease within the first two years but can remain at risk for their lifetime [22]. In the majority of immunocompetent individuals, TB infection is contained by host defense mechanisms, resulting in latent TB infection (LTBI). Tumor necrosis factor alpha (TNF-α) plays a major role in the host response to TB and confinement of the mycobacteria by the formation of the granuloma [23]. Moreover, IL-17/IL-23 axis is crucial in the immune response to Mycobacterium infections both in the innate and in the adaptive immune response, stimulating the production of antimicrobial peptides and the induction of protective T-cells [24]. In fact, chronic immunosuppression is a well-known risk factor for allowing latent tuberculosis (TB) infection to transform into active TB [25]. Therefore, before starting an immunosuppressive therapy to treat psoriasis, it is critical that the patient is screened for latent tuberculosis infection (LTBI). The patient should undergo at least an interferon–gamma release assay (IGRA) or tuberculin skin test (TST) and their clinical history and physical examination should be investigated in full. If the screening laboratory tests result is positive, further medical evaluation is required, with a chest X-ray or computed-tomography scan. If the chest X-ray results negative, the diagnosis is of LTBI it is recommended to start either 9 months of isoniazid 5 mg/kg daily with pyridoxine or 3 months of isoniazid 5 mg/kg daily plus rifampin 10 mg/kg daily plus pyridoxine with the objective of starting the biologic therapy only after completion of 1 to 2 month of prophylaxis treatment [25,26]. Additional clinical investigation should be conducted if symptoms or signs suggestive of TB, or new exposure to TB or continued residence in a high-incidence setting should occur, including a repeat interferon–gamma release assay in previously negative patients [25]. If a patient develops active TB while taking immune suppressant drugs, the immunosuppressant treatment should be stopped and the standard therapy for tuberculosis should be started [27].

#### 3.2.1. ANTI-TNFα

TNF-a plays a fundamental role in the macrophage response and in the tubercular granuloma formation and the use of TNF-a inhibitors (TNFi) in patients with psoriasis has been associated with tuberculosis reactivation or disease worsening [28,29]. In addition, atypical presentations of TB, such as disseminated and extrapulmonary disease, are more common in the setting of treatment with anti-TNF-a, as it has been reported by Cortez de Almeida et al. [30,31]. They presented a case series of two patients, who tested negative to TST screening test, showing disseminated TB after adalimumab treatment. They both reached complete resolution after having suspended TNFi treatment and having completed the anti-tubercolosis standard therapy [31]. Moreover, Cantini et al. demonstrated that patients with LTBI receiving TNFi therapy for rheumatoid arthritis or psoriasis have approximately a fourfold increase in the risk of developing active TB compared with control patients [32]. In a three-year French study, Tubach et al. highlighted that the risk of tuberculosis is three to four times higher with the use of monoclonal antibodies (MAbs), such as adalimumab, infliximab and certolizumab, compared with etanercept [33]. In fact, MAbs can induce apoptosis in TNF-a-expressing T-cells by the cross-linking of membrane-associated TNF-a and through complement- or anti- body-dependent cell-mediated cytotoxicity [26]. As shown in a study collecting the results of 18 controlled trials among a total of 3723 patients with psoriasis, cases of tuberculosis were found in 16 cases [34]. Of these, 7 cases of LTBI and 9 cases of active tuberculosis were found [34]. A pooled analysis of controlled trials for numerous conditions highlighted only one case of tuberculosis in patients on certolizumab [35]. A study comparing drugs for dermatologic and rheumatologic conditions with infliximab revealed that between 1998 and 2001, a total of 70 cases of active tuberculosis after treatment initiation were reported in the FDA’s Adverse Event Reporting System FAERS [28]. Today TNFi is considered a last-line option for the treatment of tuberculosis given its increased risk of serious infection and the availability of safer options. In patients with LTBI at least 1 month of treatment to address it is recommended before initiating the therapy.

#### 3.2.2. ANTI-IL-23

The IL-23 selective inhibitors that are approved by the US Food and Drug Administration to treat plaque psoriasis in adults are guselkumab, tildrakizumab, and risankizumab. In a two pooled study of phase 3 assessing the safety of guselkumab and anti-tuberculosis treatment (VOYAGE 1 and VOYAGE 2), no cases of active TB, including reactivation of LTBI, were reported in patients with or without LTBI treated for up to 2 years [36,37]. No cases of tuberculosis were reported in 534 patients treated with guselkumab included in the ECLIPSE trial [38]. Blauvelt et al. did not register any cases of tuberculosis in a clinical trial about continuous risankizumab therapy [39]. Moreover, in a study by Huang et al. 31 patients tested positive for LTBI by IGRA test and were left untreated for the infection while under therapy with risankizumab [40]. None of these patients developed tuberculosis reactivation during 55 weeks of follow-up [40]. In addition, no cases of tuberculosis were highlighted in two phase 3 tildrakizumab clinical trials [41]. Since low rates of tuberculosis reactivation have been reported and their favorable long-term safety profiles also in association to LTBI infection treatment, IL-23 inhibitors are considered a first-choice approach for the treatment of plaque psoriasis.

#### 3.2.3. ANTI-IL-17

So far, no cases of LTBI reactivation have been reported with IL-17-blockers. Therefore, IL17A-inhibitors are considered a safe choice to treat patients affected by psoriasis with LTBI [21,42]. In several studies, it was shown that during secukinumab treatment there were no cases of opportunistic or active TB, as in either new infections or reactivations of LTBI [43,44]. In an analysis of five phase 3 studies assessing the safety profile of secukinumab, 107 subjects out of 132 received anti-tuberculosis medication after a positive IGRA test, and 25 patients that had a negative test received secukinumab without previous anti-tuberculosis treatment [45]. No cases of active TB were reported in either group [45]. Yamaguchi et al. did not highlight any case of active TB in 129 Japanese patients treated with brodalumab for 64 weeks [46]. In addition, in the long-term extension of the phase 3 clinical trials, UNCOVER-1 and UNCOVER-2, no cases of TB reactivation were diagnosed in patients on Ixekizumab. However, one case of ‘de novo’ LTBI was reported, and the patient completed the trial after being treated with isoniazid [47].

#### 3.2.4. ANTI-IL12/IL23

The IL-12 and IL-23 immuno-response pathway is important for host protection against infections and intracellular pathogens. Ustekinumab targets the shared p40 subunit of IL-12 and IL-23 cytokines and was approved to treat plaque psoriasis. Tsai et al. reported 1 case of latent TB reactivation in a patient with psoriasis receiving ustekinumab without concomitant isoniazid treatment [48]. In 5 phase 3 trials of 3177 patients treated with ustekinumab, 167 subjects were LTBI+ and were treated concomitantly with isoniazid prophylaxis. No TB reactivation was observed, and isoniazid was well-tolerated [48]. From the analysis of a national database in South Korea, it was shown that ustekinumab did not increase the risk of TB compared to that among the general population in a real-world clinical setting [49]. In addition, Hsiao et al. reported no cases of TB reactivation in 134 patients treated with ustekinumab [50].

#### 3.2.5. ANTI-PDE4

Phosphodiesterase-4 (PDE4) is the major enzyme class responsible for the hydrolysis of cyclic adenosine monophosphate (cAMP), an intracellular second messenger that controls a network of pro-inflammatory and anti-inflammatory mediators [51]. Apremilast is a drug marketed for the treatment of psoriasis and psoriatic arthritis that, by the inhibition of the breakdown of cyclic adenosine monophosphate (cAMP), results in decreased production of inflammatory cytokines [5]. Serious opportunistic infections were comparable between placebo and apremilast in trials and no cases of LTBI reactivation have been showed so far in patients treated with apremilast [51,52]. Hagberg et al. [53] studied the risk of infections and TB with apremilast compared to DMARDs and biological drugs to treat psoriasis, showing that the incidence rates were low with the PDE4-inhibitor (0.2 per 1000 person-year).

#### 3.2.6. ANTI-JAK

The Janus kinase (JAK)-signal transducer and activator of transcription (STAT) signaling pathway plays critical roles in modulating the immune system [54]. Inhibition of Janus kinase 3 (JAK3) in T-cells is expected to block T-cell receptor-triggered signaling from downstream events and could be effective in T-cell-mediated disorders such as psoriasis [55]. Tofacitinib, by blocking the Jak-signaling, has a direct impact on dysregulated keratinocytes leading to a reduction in inflammatory infiltrates and normalization of the interleukin (IL)-23/Th17 axis [56]. The efficacy and safety of tofacitinib, the only drug in the class approved for the treatment of moderate-severe plaque psoriasis, have been demonstrated in a phase III study [54]. In an integrated long-term phase 3 study involving 6194 patients treated for up to 8.5 years with tofacitinib it was shown that 36 patients developed TB, giving an overall incidence rate of 0.2 (95% CI: 0.1–0.3) per 100 person-years [57]. In phase III studies, 263 patients diagnosed with LTBI at screening were treated with isoniazid concurrently with tofacitinib and none developed active TB [58].

### 3.3. HIV

It is known that people affected by HIV suffer a higher incidence of skin disorders, often associated with elevated morbidity [11]. Most notably, psoriasis affects HIV patients much more acutely than the general population, and usually for a longer period of time [11]. Moreover, the treatment choice for psoriasis in HIV infection is limited by systemic immunosuppression associated with some systemic and biological treatment regimens [59]. As is known, the pathogenesis of psoriasis is associated with the activation of T-cells and the treatments that reduce the T-cell count improve psoriasis. On the other hand, HIV infection is characterized by a progressive decrease in CD4+ T-cell count, and it could seem paradoxical that psoriasis exacerbations are more frequent in this subset of patients than the general population [60]; however, when psoriasis is associated with HIV infection, it is more severe due to the low control of the immune response: it was shown that with a CD4+ T-cell count <200/μL, there is a risk 9-fold greater of a severe form [61]. Moreover, HIV infection is characterized by an increased CD8+ T-cell number with an inverted CD4+/CD8+ ratio suggestive of a chronic inflammatory status, which could lead to the onset of psoriasis [61]. In fact, these patients should be managed in collaboration with infectious disease specialists for close monitoring of plasma viral load and CD4+ T-cell count. As preliminary screening for the use of immunosuppressant drugs for the treatment of psoriasis, an HIV serological testing with fourth generation enzyme-linked immunosorbent assay (ELISA) method combining the research for both antibodies and p24 antigen should be performed. If positive, HIV infection should be confirmed by either wester-blotting or NAT. The staging of the infection includes CD4 cells count and plasma HIV-RNA. Biological drugs should not be administered if the CD4+ T-cell count is lower than 200/μL [21].

#### 3.3.1. ANTI-TNF-a

The use of TNF-a inhibitors in HIV patients has been associated with an increased risk of opportunistic infections [62]. In a study by Cepeda et al. [63], 8 patients were treated with etanercept or infliximab and none of them developed either opportunistic or infectious diseases. Moreover, the CD4+ T-cell count remained stable. Adalimumab was also shown to have a good safety profile in a study by Lindsey et al. [64], with increment of the CD4+ T-cell count. In a study evaluating the use of etanercept in HIV-1-associated tuberculosis, there were no increased infectious complications in those 16 HIV-infected individuals receiving etanercept compared with the 42 HIV-infected control patients [65]. In an Italian experience by Bardazzi et al. [66], none of the 8 patients treated with TNF-a inhibitors (6 with etanercept and 2 with adalimumab) developed infectious diseases or had to interrupt the treatment because of severe immune suppression [66]. All the patients reached PASI 75% after 3 months and showed overall stable levels of CD4+ T-cell count. The data presented allow to deem TNFis as safe in the setting of HIV infection, when respecting the contra-indication of a CD-4+ T-cell count lower than 200 cells/µL.

#### 3.3.2. ANTI-IL-23

There are few published studies regarding the use of IL-23 inhibitors in HIV patients. Bartos et al. [67] did not report any adverse events in one HIV-patient successfully treated with guselkumab with CD4+ counts slightly decreased after 1 year.

#### 3.3.3. ANTI-IL-17

The literature evaluating the treatment with secukinumab of patients who are HIV-positive is limited. In a case report by Di Lernia et al. [68], a 48-year-old HIV-positive woman was successfully treated with secukinumab, with no adverse reports reported, and remained free of psoriatic lesion at follow-up visit at 12 months. Moreover, in a case series by Pangilinan et al. [69], 2 HIV-positive patients affected by erythrodermic psoriasis were treated with secukinumab and ixekizumab and registered a great improvement of PASI with no adverse events occurrences [69]. However, the well-known risk of fungal infections related to IL-17 use limits their use in immunodeficient patients, such as those who are HIV-affected [59].

#### 3.3.4. ANTI-IL12/IL23

There are numerous reports of successful administration of ustekinumab in HIV-positive patients affected by psoriasis [66,70]. Bardazzi et al. treated 4 patients for a mean duration therapy of 34.8 months with ustekinumab and adverse events were not reported, including serious and opportunistic infections [66]. It is demonstrated that the CD4+ T-cell count as well as the viral load not only remain stable, but also improve in some cases [66,71]. Ustekinumab not only shows a good safety profile but also seems to achieve good therapeutic outcomes, with an improvement of PASI and patients’ quality of life, making it among the first-line drugs [66]

#### 3.3.5. ANTI-PDE4

Several case reports have been published recently regarding the use of apremilast in HIV patients affected by psoriasis [72,73,74,75]. Neither opportunistic nor serious infectious disease were reported. Moreover, apremilast was shown to be safe and succesful in complex patients co-infected with HIV and either HCV or HBV [74,75]. Due to its “low” immunosuppressive properties, apremilast should be considered a first-line treatment in HIV patients. 

#### 3.3.6. ANTI-JAK

No data were published during the last three years about the use of tofacitinib in patients with HIV infection and psoriasis.

### 3.4. HBV

While the association between psoriasis and hepatitis C has been demonstrated, hepatitis B risk of infection seems not to be correlated to psoriasis [76,77]. In a multivariate analysis by Cohen et al. [76] it was found that the prevalence of psoriasis was not significantly associated with hepatitis B (OR = 1.22, 95% CI = 0.93–1.60). Before starting a systemic therapy with immune suppressive drugs, patients must be screened for hepatitis B virus (HBV) infection, looking for the presence of antibodies directed against HBV core (HBcAb) and HBV surface antigen (HBsAb). Moreover, HBV surface antigen (HBsAg) should be searched. If HBsAg results are positive, with the absence of HBsAb, it indicates that the patient has either recently been infected with HBV or at all [78]. Plasma HBV-DNA should be measured and treatment for the infection should be started. If HBcAb is found positive, in absence of HBsAg, this is called “occult hepatitis B” [78]. It must be determined if we are in presence of an active or inactive carrier by measuring plasma HBV-DNA, quantitative HBsAg, HBeAg, HBeAb (IgG). When plasma HBV-DNA is detectable, treatment with entecavir or tenofovir should be started before biological treatment [78]. Biological treatment should be initiated when plasma HBV-DNA is either undetectable or at least lower than 2000 UI/mL [79]. If plasma HBV-DNA is not detectable, it is recommended to start lamivudine (100 mg/daily) 2 weeks before biological drugs and continue up to the end of therapy. HBcAb appears with acute infection and persists for life, prophylaxis is not required when Abs are the only sign of the infection, but clinical and laboratory monitoring is recommended [79]. The isolated presence of HBsAb indicates either recovery or immunity because of vaccination and no measures are required [21].

#### 3.4.1. ANTI-TNF-a

TNF-α plays an important role in the clearance of hepatitis B virus from infected hepatocytes, therefore TNFi may lead to hepatitis reactivation or disease worsening. An analysis of 257 HBsAg or anti-HBc-positive patient receiving TNFi showed HBV reactivation in 39% of HBsAg positive patients compared with 5% of isolated anti-HBc positive patients [80]. HBV reactivation was more frequent in patients who did not undergo antiviral prophylaxis compared with patients who did (62% vs. 23%). Patients with HBcAb positivity pose a lower risk of viral reactivation compared with patients with HBsAg positivity [80]. All the molecules belonging to TNFα inhibitors have been shown to be associated with drug-induced liver injury; therefore, it is highly recommended to monitor the liver enzymes during the therapy [81]. If the patient is HBsAg+/anti-HBc+, antiviral prophylaxis should be commenced concomitantly or 1–2 weeks before TNFi therapy is initiated. The duration of treatment should be decided with the hepatologist [80]. On the other side, when the patient is HBsAg-/anti-HBc-, vaccination should be considered 2 weeks before starting biological therapy. However, a consultation with hepatologist and a triple serology screening with LFTs is highly recommended before starting the TNFi therapy in order to have a serologic risk stratification between nonimmune, immune due to vaccination, resolved previous hepatitis infection, acute infection, chronic infection, and occult infection and decide an appropriate initiation of antiviral prophylaxis and/or vaccination.

#### 3.4.2. ANTI-IL12/23

The role of IL-23 in immune response to hepatotropic viruses is still unclear [82]. According to literature, only two cases of patients (one pediatric), both treated with ustekinumab and then guselkumab and having respectively HBsAg negativity and HBsAg/anti-HBcAg positivity have been reported. Of note, the pediatric case had most likely acquired HBV and was considered to be in a chronic inactive carrier state. In both cases, no reactivation occurred, liver enzymes remained stable, and HBV-DNA of pediatric patient progressively decreased to become undetectable [82,83]. Ustekinumab seems to be associated with a moderate risk of reactivation, according to the few data available in literature (10 studies, 3 patients with HBV reactivation, one also using MTX, only one receiving prophylaxis with lamivudine) [84].

#### 3.4.3. ANTI-IL17

The hypothesis of a possible beneficial role of Il-17 inhibitors on the development of liver fibrosis still needs to be confirmed [84]. Regarding the real-world data, few cases have been reported. In one prospective study performed on 49 HBV patients, only 4 reactivations were reported out of 22 HBsAg+ treated patients and only one out of 24 in HBsAg-/anti-HBc+ subjects. No reactivation was found in the three remaining HBsAg+ patients receiving antiviral treatment [85]. Cases of chronic or resolved HBV infection in patients undergoing secukinumab therapy have been reported [86,87], but none were found to have hepatitis or virus reactivation. A good safety profile was shown also for ixekizumab: 2 reports in the literature (one with active HBV infection and concurrently treated with entecavir and one with both markers of past HBV infection and anti-HCV positivity and no signs of reactivation) suggest the drug as safe in this setting [88,89]. No cases of reactivation of HBV have been reported in randomized controlled trials of brodalumab and its safety has not been yet established in HBV or HCV patients by real-world data [84].

#### 3.4.4. ANTI-PDE4

Given the mechanism of action, apremilast represents a safe option in conditions in which immunosuppression is contraindicated and has no hepatotoxic effects [90]. In addition, the anti-fibrotic action showed in animal models by inhibiting pro-fibrotic factors such as TGFβ and IL-13, highlighted a potentially positive impact on liver fibrosis [84] Only one case in literature has been described of a patient with chronic viral hepatitis B treated with apremilast with no concomitant antiviral treatment [75]. The liver function tests consistently remained within normal ranges after 1 year, and HBV-DNA was always negative.

#### 3.4.5. ANTI-JAK

No data were published during the last three years about the use of tofacitinib in patients with HBV infection and psoriasis.

### 3.5. HCV

Several studies showed a higher prevalence of HCV infection in patients with psoriasis [76,77,91]. In a large cohort study by Cohen et al. [76] among 12.000 patients, it was shown that the prevalence of hepatitis C in psoriatic patients was 1.03%, compared to 0.56% in controls. This could be explained by the increased cutaneous levels of cathelicidin, TLR9, and IFNc of HCV-positive psoriatic patients in comparison to HCV-negative psoriatics, suggesting that HCV infection may predispose patients to developing psoriasis [92]. HCV infection is diagnosed when anti-HCV antibodies are detected. A patient with a positive result must undergo HCV-RNA testing, or HCV-core-antigen testing whenever HCV-RNA assays are uunavailable or not affordable, to diagnose an active infection [93]. If HCV-RNA is positive, it is mandatory to test liver functions and to start antiviral treatment with direct-acting antivirals (DAA), which are effective in eradicating the infection; biological therapies should not be commenced if cirrhosis is not controlled [79]. Unlike HBV reactivation, HCV reactivation is quite uncommon, occurring primarily with immunosuppressive drugs administered to chronically infected patients who were not carefully evaluated and did not receive antiviral therapy [94]. 

#### 3.5.1. ANTI-TNF-a

The suppression of TNF-α by biological agents was considered to be a possible threat to viral replication and possible chronic HCV infection. On the contrary, a high production of TNF-α was found in patients chronic HCV infections, with implications for liver injury [95]. Among TNFi, the use of etanercept seems to be safer, as it is a less potent inhibitor of TNF-α activity and less able to induce complement-dependent cytotoxicity and transmembrane TNF-α apoptosis [5]. Moreover, the antiviral effects of interferon and ribavirin were increased by etanercept in treatment-naïve patients with chronic HCV infection that presented a high sustained virologic response and a low frequency of adverse events [96]. On the other side, the progression of the cirrhosis and hepatocellular carcinoma could potentially be accelerated through the immunosuppression induced by TNF-α-inhibitors [5]. Di Nuzzo et al. [97] reported two cases of hepatocellular carcinoma that developed in HCV patients with psoriasis with the cirrhotic disease during long-term etanercept treatment. In addition, adalimumab showed a safe profile in HCV-positive individuals affected by psoriasis. In a recent study on 20 HCV patients with psoriasis treated with adalimumab for a median of 40 months, the log-rise of plasma HCV viral load was found only in 3 patients. The increase was not associated with concurrent hepatic cytolysis or cholestasis indexes and none of the patients reached the criteria for HCV reactivation [98]. To summarize, caution should be used in the administration of TNFα inhibitors in compensated patients, who need to be monitored by ultrasound imaging; but they are not suitable for patients with decompensated liver disease because of their increased risk of potentially serious infections [97].

#### 3.5.2. ANTI-IL-23 

Data on the safety of IL-23 inhibitors are limited, although their profile appears to be favorable [84]. 

#### 3.5.3. ANTI-IL-17

Studies investigating the safety profile of IL-17 inhibitors in the context of HCV infection are limited to small case series with a short follow-up. In a study by Chiu et al. evaluating the safety profile of secukinumab among patients affected by psoriasis and concomitant HBV or HCV, one of 14 (7.1%) patients reported increased HCV replication [85]. On the other hand, a patient with psoriasis and concomitant HBV-HCV infection was successfully treated with secukinumab and no adverse effects or reactivation were reported [86]. Overall, because robust data on the safety of secukinumab on HCV are lacking, it seems wise to consider patients with HCV infection receiving secukinumab as those who are treated with TNF-α inhibitors [84].

#### 3.5.4. ANTI-IL12/IL23

Limited data are available on patients with concurrent HCV infections treated with ustekinumab. In a study by Chiu et al. [99] evaluating 4 HCV patients affected by psoriasis under ustekinumab treatment, there was one reactivation and two cases of a slight rise in HCV-RNA. In two case reports, no reactivation was reported in psoriatic patients with concomitant HCV treated with ustekinumab. Moreover, long-term remission was maintained [100,101]. 

#### 3.5.5. ANTI-PDE4

Few cases of HCV patients affected by psoriasis and treated with apremilast are described in the literature [74,102]. No cases of reactivation or elevation of liver enzymes are reported. The molecule is considered a safe option for patients with several conditions, such as active cancer or infection, for which conventional immunosuppressive therapy is contraindicated [90]. 

#### 3.5.6. ANTI-JAK

No data were published during the last three years about the use of tofacitinib in patients with HCV infection and psoriasis.

### 3.6. Hospital-Acquired Infections

Hospital-acquired infections (HAIs) are more and more important on the scene of infectious diseases [19]. Although psoriasis has been shown to be an independent risk factor for serious and opportunistic infections, it has also been demonstrated that the introduction of a treatment with biologic drugs increases the risk of infection of about 55% [15,19,103]. Moreover, the intravenous administration and the use of port-a-cath, increase the chance of developing bloodstream infections whenever the personnel handling the device has not been correctly trained [104]. An increased rate of HAIs is also correlated with the advanced age of the patients and hospital admission frequency [19]. In addition, psoriasis alone doubles the hospitalization rate for infection [19]. This could be also related to the fact that psoriatic patients are shown to have a 2.5-fold risk of developing chronic obstructive pulmonary disease and diabetes [19]. Moreover, IL-17 and IL-23 are pro-inflammatory cytokines involved in the defense of extracellular bacteria and fungi. Therefore, when IL-17 production is decreased, recurrent infections with Staphylococcus aureus and Candida albicans might be seen. Phase 2 and 3 studies on anti-IL-17 and anti-IL-23 did not show any increased risk of serious infections [105,106]; however, post-marketing studies highlighted an increased risk of opportunistic infections such as esophageal candidiasis, herpes zoster, pneumonia, and even Mycobacterium avium complex infections, especially with the use of anti-IL-23 drugs [107]. Hence, it is of utmost importance to attentively evaluate each patient for their infectious risk before beginning a treatment.

### 3.7. COVID-19

Whether psoriasis itself could confer susceptibility to SARS-CoV-2 virus infection is not known [108,109]. What is known is that systemic treatments, including synthetic or biologic disease-modifying antirheumatic drugs (DMARDs), have been associated with increased risk of infection, including respiratory tract viral infection [110,111]. In addition, patients with moderate-to-severe psoriasis are frequently affected by cardiometabolic comorbidities, such as obesity, diabetes mellitus, and hypertension, all representing negative prognostic factors for pneumonia by SARS-CoV-2 infection [112]. For these reasons, initiation of biologic therapy is not recommended in active SARS-CoV-2 infection and the transition to safer alternatives may be considered to avoid serious complications [113]. Several studies have assessed the incidence of SARS-CoV-2 infection in patients with psoriasis receiving systemic biological treatments and, apparently, there is no increased susceptibility to SARS-CoV-2 infection [112,114,115]. Studies have also been made reporting the risk of hospitalization, intensive care unit admission, and mortality due to COVID-19 in psoriasis patients treated with systemic therapies. No hospitalization or death was documented in 980 patients with psoriasis on biologics in a large cohort study by Gisondi et al. [116]. Moreover, several case reports and retrospective studies have been made about the course of COVID-19 in psoriasis patients receiving biological therapies [114,117,118,119]. In all the cases, full recovery from COVID-19 was reported after biologic treatment interruption. Favorable outcomes and no severe adverse effects were registered in patients that did not interrupt the biologic treatment, such as adalimumab and ustekinumab [118,119]. Other studies have shown that in presence of severe viral symptoms with high fever develop, the discontinuation of IL-17 inhibitors should be considered [120,121]. On the other hand, although IL-17 has an important role in mucosal immunity, preliminary studies by Wan et al. [122] and Tathiparthi et al. [123] did not prove any increased risk of either severe infection or negative outcome. Moreover, apremilast was shown having a good safety profile in COVID-19 affected patients, in fact, it does not favor either infection or cytokine storm, and it does not increase the risk of pulmonary fibrosis [124]. Factors to consider when selecting systemic treatment during COVID19-infection are summarized in Table 2.

## 4. Conclusions

The presence and/or the possibility of comorbid conditions is fundamental when planning treatment and overall management of psoriatic patients, especially those with moderate-to-severe forms of the disease. With regard to infections, psoriasis itself represents a major risk factor. Moreover, the risk of concomitant infections may be increased by the administration of certain immunosuppressive drugs, so patients with psoriasis are recommended to be carefully screened for possible latent systemic infections. New emerging conditions, including the COVID-19 pandemic, have further highlighted the need to take into account the risk of infection in approaching such patients. According to the latest literature data and registers, using novel therapies, particularly anti-ILs and anti-PDE4, seem not to have a significant impact on the vulnerability of these patients to infections, thus representing a reassuring option in the management of the disease. 

Attention must, however, be kept high given the extensive use of these drugs, the presence of several comorbidities as well as the recrudescence of ‘ancient’ diseases, and the emergence of new infectious agents.

## Figures and Tables

**Table 1:** Factors to consider when selecting systemic treatment during infections.

Class of Drugs	Drug	TBC	HIV	HBV	HCV
TNF-a inhibitors	Etanercept	+	++	+	++
	Adalimumab	- +	++	+	+
	Infliximab	- +	++	+	+
	Certolizumab	- +	++	+	+
IL-23 inhibitors	Guselkumab	++	?+	?+	?+
	Tildrakizumab	++	?+	?+	?+
	Risankizumab	++	?+	?+	?+
IL-17 inhibitors	Secukinumab	++	- +	?+	?+
	Ixekizumab	++	- +	?+	?+
	Brodalumab	++	- +	?+	?+
IL-12/23 inhibitors	Ustekinumab	++	++	?+	?+
PDE4 inhibitors	Apremilast	++	++	?+	?+
JAK inhibitors	Tofacitinib	+	?	?	?

Note: Two plus symbols (++) indicates preferred agents; one plus syndrome (+) indicates that the agent can be used; one plus symbol and one minus symbol (+ -) indicate that the drug can be used but is controversial; one minus symbol and one plus symbol (- +) indicate that the drug is not preferred but can be used; one question mark and one plus symbol (?+) indicate that there are not enough data but the drug is likely safe to use; one question mark (?) indicates that there are not enough data; one minus symbol (-) indicates that use of that drug is controversial because there are not enough data; and (x) indicates that the drug is contraindicated.

**Table 2:** Factors to consider when selecting systemic treatment during COVID19-infection.

Class of Drugs	Drug	COVID-19
TNF-a inhibitors	Etanercept	++
	Adalimumab	++
	Infliximab	++
	Certolizumab	++
IL-23 inhibitors	Guselkumab	++
	Tildrakizumab	++
	Risankizumab	++
IL-17 inhibitors	Secukinumab	- +
	Ixekizumab	- +
	Brodalumab	- +
IL-12/23 inhibitors	Ustekinumab	++
PDE4 inhibitors	Apremilast	++
JAK inhibitors	Tofacitinib	?

Note: Two plus symbols (++) indicates preferred agents; one plus syndrome (+) indicates that the agent can be used; one plus symbol and one minus symbol (+ -) indicate that the drug can be used but is controversial; one minus symbol and one plus symbol (- +) indicate that the drug is not preferred but can be used; one question mark and one plus symbol (?+) indicate that there are not enough data but the drug is likely safe to use; one question mark (?) indicates that there are not enough data; one minus symbol (-) indicates that use of that drug is controversial because there are not enough data; and (x) indicates that the drug is contraindicated.

## Data Availability

Not applicable.
